# A protocol for obtaining upper and lower extremity joints’ range of motion in children using three-dimensional motion analysis system

**DOI:** 10.3389/fphys.2024.1416175

**Published:** 2024-08-21

**Authors:** Mohamed Afifi, Muhammad Uba Abdulazeez, Kamiar Aminian, Georgios Antoniou Stylianides, Kassim Abdulrahman Abdullah

**Affiliations:** ^1^ Department of Mechanical and Aerospace Engineering, College of Engineering, United Arab Emirates University, Al Ain, United Arab Emirates; ^2^ Emirates Center for Mobility Research, United Arab Emirates University, Al Ain, United Arab Emirates; ^3^ Department of Automotive Engineering, Faculty of Engineering and Engineering Technology, Abubakar Tafawa Balewa University, Bauchi, Nigeria; ^4^ Laboratory of Movement Analysis and Measurement, École Polytechnique Fédérale de Lausanne, Lausanne, Switzerland; ^5^ Exercise Science and Kinesiology, Juniata College, Huntingdon, PA, United States

**Keywords:** joint ROM, extremity, 3D motion analysis, joint coordinate system, marker placement model

## Abstract

Three-dimensional (3D) motion analysis (MA) techniques are progressively being used in biomechanics research and for clinical applications to assess the risk of injuries. A marker-based 3D MA protocol has been developed to measure the upper and lower extremity (UE and LE) joints’ active and passive ranges of motion (AROM and PROM) in children. The joints that were included in this protocol are shoulder, elbow, wrist, hip, knee and ankle. The anatomical joint coordinate systems (JCS) have been defined for the upper and lower extremities to standardize reporting. A marker placement model was defined according to the International Society of Biomechanics (ISB) recommendations and used to develop the protocol. The proposed movements will be captured and analyzed using the Motion Analysis Corporations 3D MA system integrated with Cortex software. The movements adopted in this study have been selected from various sources to incorporate all joint rotations while ensuring the isolation of each joint motion during the movements. It is recommended that future studies utilize this protocol to draw a relationship between the joints’ range of motion (ROM) and the adjacent segments characteristics, i.e., segment length, joint stiffness, etc.

## 1 Introduction

A variety of techniques and instruments have been utilized to measure the UE and LE joints ROM. When a joint’s AROM and PROM are under assessment, it is crucial that no contraindications to the assessment procedures exist, such as pain due to an inflammation in a joint or the region around a joint, or after an injury where there has been damage of soft-tissue (i.e., muscle, tendon, ligament, etc.) (Clarkson, 2020). Performing AROM and PROM assessments in the presence of pain will not only restrict achieving the ROM but could also disrupt the healing process or result in the deterioration of the condition. Measuring the PROM is a common clinical assessment among adults and children. The point at which the maximum ROM is reached during passive assessment is typically described as the end-feel, which acts as a barrier to further motion as slight overpressure is applied (Eastman et al., 2022). Certain joints are structured such that the joint capsules limit the end of the particular ROM, while other joints are structured so that ligaments limit the end of the ROM (Norkin and White, 2016).

3D MA techniques using marker based stereophotogrammetry are progressively being used in biomechanics research and for clinical applications to assess the risk of injuries (Ugbolue et al., 2021; Reissner et al., 2019). It has been recommended that concurrent validity tests are included in studies aiming to investigate ROM measurements using 3D MA and manual goniometry. 3D MA is yet considered the “gold standard” for quantifying dynamic motion in research and in clinical practice (Ore et al., 2020). The validity of 3D MA was thoroughly assessed by comparing it with advanced radiography in a previous study (Miranda et al., 2013), which concluded that it is possible to measure “true” joint motion in dynamic activities using 3D MA. Several studies have also reported the validity and the repeatability of using 3D MA systems to measure the joints’ ROM (Ore et al., 2020; Reissner et al., 2019; Lempereur et al., 2014; Tojima et al., 2013). These studies have provided a comparison between marker-based 3D MA and the conventional goniometry to measure the ROM of various joints of the body.

Injuries in both the lower and upper extremities of the child’s body are the second most frequent injuries sustained by children in vehicle crashes worldwide (Hanna, 2010; Jermakian et al., 2007; Fildes et al., 1997). Children usually sustain upper extremity injuries due to airbag deployment and adult seatbelt use (García-España and Durbin, 2008; Arbogast et al., 2003). These children are 2.5 times more likely to sustain upper extremity injuries compared to other children in similar crashes that are not exposed to the deploying airbag. The injuries sustained are usually to the forearm, finger, hand, clavicle, and elbow with girls twice as likely to sustain these injuries compared to boys. Upper extremity joint dislocation has been identified as one of the factors contributing to long-term disability in children due to contact with deploying airbags (Jernigan et al., 2005). Additionally, the extremity injury outcomes in children are worse than that of adults in similar crashes due to their developing body (Hanna, 2010).

The rear seat had been established to be the safest sitting position for children and about seventy-nine percent of rear seat occupants globally are children less than 12 years old (Trowbridge and Kent, 2009; Durbin and Hoffman, 2018). However, despite the obvious safety benefits of riding in the rear-seat, rear-seated children are still susceptible to sustaining severe lower extremity injuries especially in frontal crashes (Trowbridge and Kent, 2009; Arbogast et al., 2004; Arbogast et al., 2002). The ankle, foot, and tibia are usually the first contact points with the vehicle interior for rear-seated children in frontal collisions (Seidel et al., 2018). Studying the kinematics of the pediatric extremity joints is essential in order to provide enhanced protection for children against lower extremity injuries in vehicle crashes.

Therefore, the aim of the present study is to develop a protocol to obtain the UE and LE joints AROM and PROM in children using a marker-based 3D optical motion capture (OMC) system, assessing 3D motion capture for ROM in children. The protocol will be used in future studies to obtain the extremity joints’ active and passive ROM in children for assessing the risk of joint injuries due to vehicle crashes and in the evaluation of extremity joint impairments due to rheumatic, neurological, musculoskeletal, and neuromuscular disorders thereby enhancing the safety outcomes of extremity injuries and treatments of joint impairments in children.

## 2 Equipment

### 2.1 Instrumentation and laboratory configuration

A marker-based 3D OMC system incorporating 12 digital cameras (Raptor-12HS, Motion Analysis Corporation, CA, United States) integrated with Cortex software (Cortex 9.0, Motion Analysis Corp. 2021) will be used to capture and analyse the movements.

### 2.2 Virtual markers (VMarkers)

The Cortex 9.0 software is equipped with a tool that facilitates the addition of VMarkers to the model. This tool generates an estimate of the joint center positions where actual markers cannot be placed. VMarkers get their position from a combination of two or three actual markers in the motion capture data.

### 2.3 Skeleton builder

Skeleton Builder is a tool within Cortex that allows the construction of skeletal bones for building the models. A skeleton is a hierarchically connected set of bones with translation and rotation data. Each bone is defined by the motion of three markers to construct its rotation data (Motion Analysis Corporation, 2022). The skeleton generates a 3D coordinate system which simplifies the assessment of the rotation of a segment about the joint center.

## 3 Methods

### 3.1 Participants

A cross-sectional study following the development of the protocol will be performed at the Motion Analysis Laboratory at United Arab Emirates University (UAEU), recruiting 191 children aged 4–12 years. The guideline provided by ISO 15535 standard (ISO, 2008) (Eq. 1) was used to establish the sample size required for this study according to the following expression:

$$n={\left(\frac{1.96\times CV}{a}\right)}^{2}\times 1.534^{2}$$ (1)

where 1.96 is the critical value from a standard normal distribution for a 95% confidence interval; CV is the coefficient of variation; *a* is the proportion of relative certainty required. Based on the values of CV provided by Pheasant and Haslegrave (2016), the sample size was obtained as follows:

$$n={\left(\frac{1.96\times 13}{2}\right)}^{2}\times 1.534^{2}=190.97=191\;subjects$$

Data will be collected from the left limbs of each participant for standardization. All participants will be informed about the procedures and instructed on how to perform the proposed movements. Ethical approval for the study has been obtained from the UAEU’s Human Ethics Research Committee (ERH_2022_1306_15). The children will be recruited from selected schools in Al Ain city, Abu Dhabi, United Arab Emirates. The inclusion criteria will include: i) aged between 4 and 12 years; ii) not having any musculoskeletal, neurological, neuromuscular, or rheumatic disorder. On the other hand, the exclusion criteria will be any impairment that can restrict normal joint flexibility. Informed consent will be obtained from the children’s parents and assent will be obtained from the participants before starting the study process.

### 3.2 Motion analysis protocol

The protocol employed in this study was developed in accordance with the framework proposed by Kontaxis et al. (2009). The guidelines presented by Kontaxis et al. summarized the essential steps required in the definition of MA protocols, aiming to support the standardisation of protocols based on general recommendations.

#### 3.2.1 Segment definitions and joint coordinate systems

The joints investigated in this study are: 1) shoulder (glenohumeral), 2) elbow (humeroulnar/humeroradial), 3) wrist (radiocarpal joint), 4) hip (iliofemoral joint), 5) knee (tibiofemoral), and 6) ankle (talocrural). Although these joints exhibit translational and rotational movements about their axis of rotation, only the rotational movement about the joint centers will be examined (referred to as the osteokinematic motion).

In order to extract the joint kinematics from the proposed movements, segments which represent different portions of the body must be defined. A segment comprises a set of markers linked together to define a body part, i.e., anterior superior iliac spine (ASIS), posterior superior iliac spine (PSIS) and the sacrum linked together form the pelvis segment. This facilitates the calculation of the rotation about three axes of one segment with respect to another. Table 1 shows the moving segments with respect to the reference segments.

**TABLE 1:** Biomechanical model segment definitions.

Designated joint	Moving segment	Reference segment
Shoulder	Arm	Scapula
Elbow	Forearm	Humerus
Wrist	Hand	Forearm (Ulna/Radius)
Hip	Thigh	Pelvis
Knee	Shank	Femur
Ankle	Foot	Tibia

**FIGURE 1:**
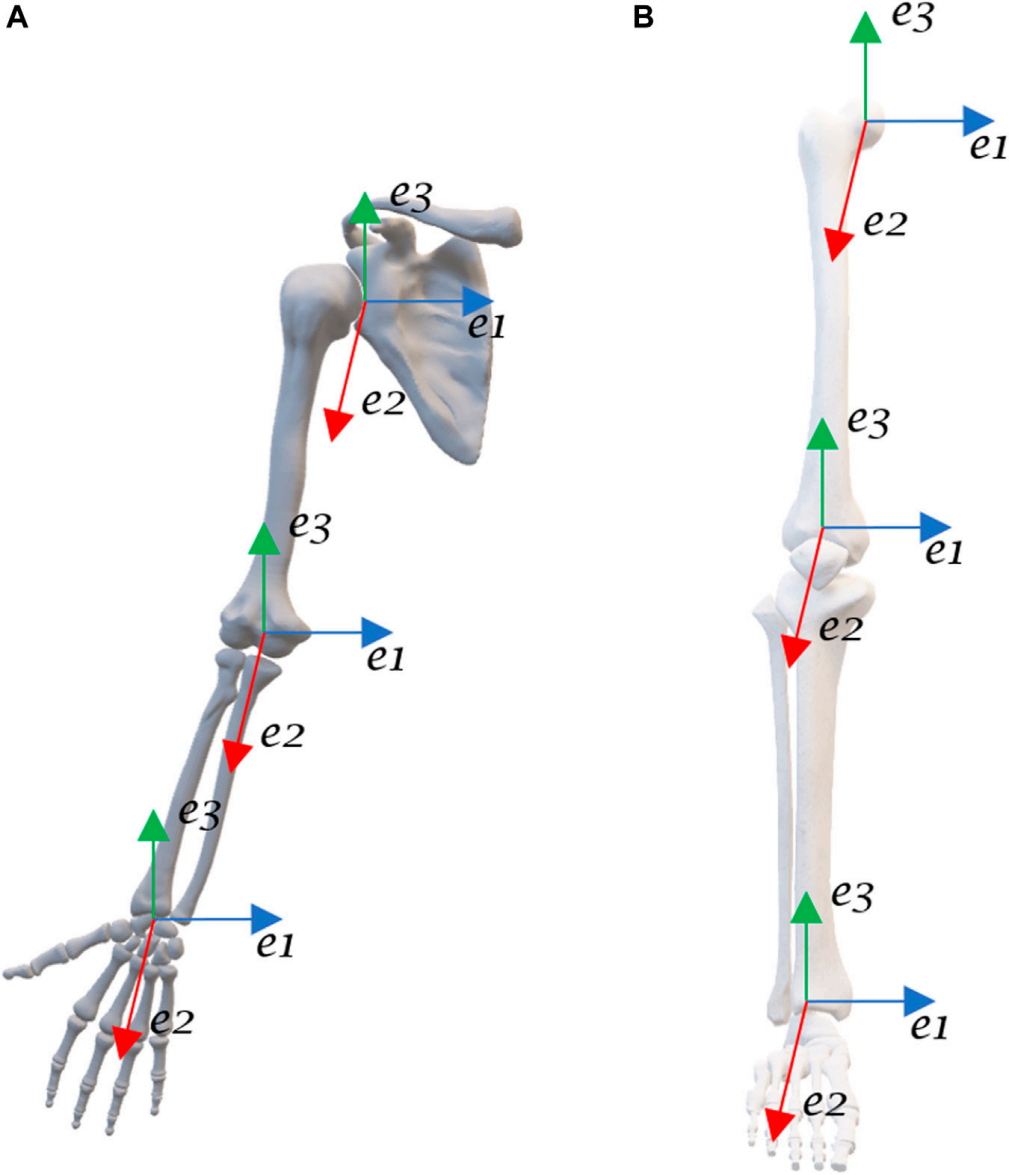
Anatomical joint coordinate systems of the **(A)** UE and **(B)** LE.

The JCS have been defined according to the ISB recommendations and Grood and Suntay (Wu et al., 2005; Wu et al., 2002; Leardini et al., 2021; Grood and Suntay, 1983) (Figure 1; Table 2). The shoulder joint is modelled as a ball and socket joint with 3 degrees-of-freedom (DOF), located at the centre of the humeral head. The shoulder flexion/extension occur in the sagittal plane about the e1-axis, adduction/abduction in the frontal plane about the e2-axis, and internal/external rotation in the transversal plane about the e3-axis. The elbow joint is modelled as a rotating hinge-joint with 2 DOF, flexion/extension in the sagittal plane occur about the e1-axis and pronation/supination of the forearm in the transversal plane about the e3-axis. The wrist joint is modelled as a universal (saddle) joint with 2 DOF, flexion/extension in the sagittal plane occur about the e1-axis and radial/ulnar deviation in the frontal plane about the e2-axis (Cutti et al., 2005; Rab et al., 2002). The axes shown in Figure 1 on the UE joints are only a simplification, refer to Table 2 for the exact definitions.

**TABLE 2:** Joint coordinate systems.

Joint	Axis		
	e1	e2	e3
Shoulder	Fixed to the scapula, line connecting the acromion (AC) to the TS.	Perpendicular to the segment formed by the GH and the EL/EM pointing forward.	Line connecting the EL/EM midpoint to the GH.
Elbow	Line connecting the EM and the LM.	Floating axis, perpendicular to e1 and e3.	Line connecting the US to the midpoint of the EM and EL.
Wrist	Line connecting the US to the RS.	Floating axis, perpendicular to e1 and e3.	The line parallel to the shaft of the radius starting from the midpoint between the US and the RS.
Hip	Perpendicular to the femoral axis (e3) pointing to the lateral/medial direction.	Floating axis, perpendicular to e1 and e3.	Axis fixed to the femur extending from the femoral head to the midpoint of the FE’s.
Knee	FE’s axis extending from the lateral FE to the medial FE.	Floating axis, perpendicular to e1 and e3.	Tibial axis extending from the tibial tuberosity to the most inferior tibia (inter-malleolar point).
Ankle	Fixed to the tibia/fibula segment extending from the lateral malleolus to the medial malleolus.	Floating axis, perpendicular to e1 and e3.	Tibial axis extending from the tibial tuberosity to the most inferior tibia (inter-malleolar point).

AC, Acromioclavicular; TS, Trigonum spinae; EL/EM, lateral/medial epicondyles of humerus; GH, Glenohumeral joint; US, Ulnar styloid; RS, Radial styloid; FE, Femoral epicondyles.

The hip joint is modelled as a ball and socket joint with 3 DOF, located at the centre of the femoral head. The hip flexion/extension occur in the sagittal plane about the e1-axis, adduction/abduction in the frontal plane about the e2-axis, and internal/external rotation in the transversal plane about the e3-axis. The knee joint is modelled as a rotating-hinge joint with 2 DOF, flexion/extension occur in the sagittal plane about the e1-axis and the internal/external rotation in the transversal plane about the e3-axis. Although the knee is subject to considerable varus-valgus load during functional activities, it is yet difficult to voluntarily generate moment about the varus-valgus axis (e2-axis), i.e., control knee abduction/adduction (Zhang et al., 2001). The ankle joint is modelled as a universal joint with 2 DOF, plantarflexion/dorsiflexion occur about the e1-axis and inversion/eversion about the e2-axis (Slater et al., 2018).

While the joint axes defined for this protocol are based on ISB recommendations, the functional axes might slightly differ from the defined axes. This is because during experimentation, markers are placed on the bony landmarks which best correspond to the defined axis. Therefore, the orientation of the resulting axis is the closest estimation to the anatomical joint axis.

#### 3.2.2 Marker placement

**FIGURE 2:**
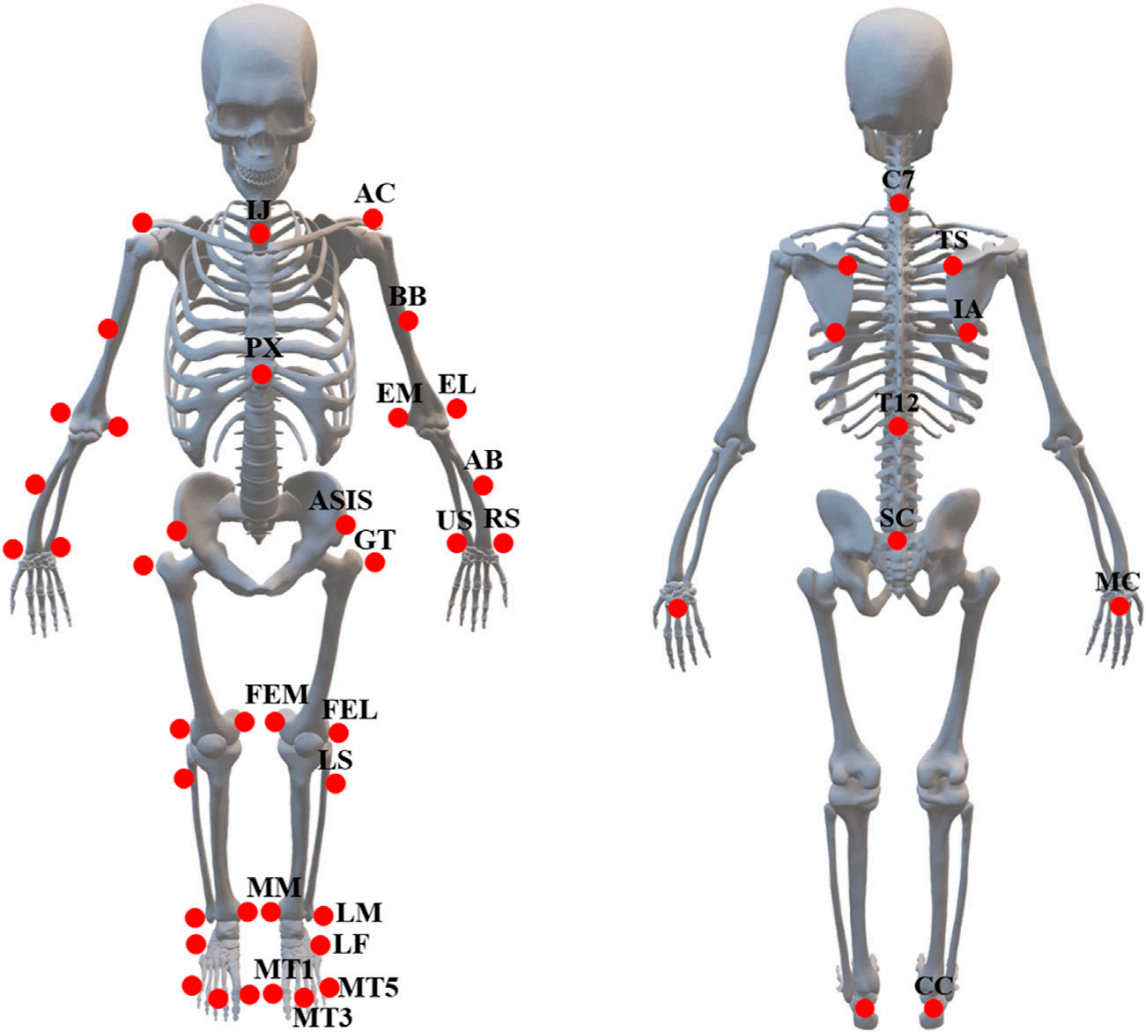
Marker placement model.

The UE and LE markers locations, shown in Table 3, have been defined with regard to ISB recommendations and Grood and Suntay (Wu et al., 2005; Wu et al., 2002; Leardini et al., 2021; Grood and Suntay, 1983) (Figure 2). The marker placement model have been modified to facilitate the definition of segments and allow marker tracking on Cortex 9.0.

**TABLE 3:** Anatomical marker locations.

Joint	Bony landmark	Description
**Shoulder**	Incisura Jugularis (IJ) (Sternal Notch)	Deepest point of the sternal notch.
	Processus Xiphoideus (PX)	Most caudal point on the sternum.
	C7	Most prominent inferior part of the cervical spine.
	Acromioclavicular (AC) (Shoulder)	Most dorsal point on the acromioclavicular joint (lateral end of the clavicle, shared with the scapula).
	Trigonum Spinae Scapulae (TS) (Upper Scapula)	Midpoint of the triangular surface on the medial border of the scapula in line with the scapular spine.
	Inferior Angle (IA) (Lower Scapula)	Most caudal point of the scapula.
	T12	Most inferior part of the thoracic spine level with the 12th rib.
	Biceps Brachii (BB) (Arm)	Large muscle on the ventral side of the arm.
**Elbow**	Lateral Epicondyle (EL)—Humerus (Lateral Elbow)	Most caudal point on the lateral epicondyle.
	Medial Epicondyle (EM)—Humerus (Medial Elbow)	Most caudal point on the medial epicondyle.
	Antebrachium (AB) (Forearm)	Ventral side of the forearm.
**Wrist**	Radial Styloid (RS) (Lateral Wrist)	Most caudal-lateral point of the radial styloid.
	Ulnar Styloid (US) (Medial Wrist)	Most caudal-medial of the ulnar styloid.
	Third Metacarpals (MC3) (Hand)	Distal head centre of the third metacarpal.
**Hip**	Anterior Superior Iliac Spine (ASIS)	Most anterior point of the iliac.
	Sacrum (SC)	Most inferior point of the lumbar spine.
	Greater Trochanter (GT)	Tip of the greater trochanter of the femur.
**Knee**	Femoral Epicondyles (FEL and FEM) (Lateral and Medial Knee)	Lateral and medial epicondyle of the femur.
	Lateral Shank (LS)	Lateral side of the tibia.
**Ankle**	Medial Malleolus (MM) (Medial Ankle)	Tip of the medial malleolus.
	Lateral Malleolus (LM) (Lateral Ankle)	Tip of the lateral malleolus.
	Calcaneus (CC) (Heel)	Most dorsal point on the calcaneus.
	Lateral Foot (LF)	Lateral side of the base of the fifth metatarsal.
	First Metatarsal (MT1)	Head of the first metatarsal.
	Third Metatarsal (MT3)	Head of the third metatarsal.
	Fifth Metatarsal (MT5)	Head of the fifth metatarsal.

### 3.3 Experimental procedures

Participants will be instructed to wear light clothing without shoes during the session. Before commencing the experimental protocol, participants will be informed of the components of the MA system and the movements involved. Several anthropometric measurements, shown in Table 4 will be recorded prior to the ROM data collection for analysis. A time of 40 min is expected for the participant’s familiarisation, the anthropometric measurements, and the marker attachment.

**TABLE 4:** Anthropometric measurements and their corresponding measuring devices.

Anthropometric measurement	Measuring device
Bideltoid breadth	Anthropometer (Seca, Hamburg, Germany)^a^
Biacromial breadth	Anthropometer (Seca, Hamburg, Germany)
Shoulder-Elbow length	Segmometer (Cescorf, Porto Alegre, Brazil)^b^
Elbow-Hand length	Segmometer (Cescorf, Porto Alegre, Brazil)
Bi-trochanter breadth	Anthropometer (Seca, Hamburg, Germany)
Buttock-Knee length	Anthropometer (Seca, Hamburg, Germany)
Knee height	Anthropometer (Seca, Hamburg, Germany)
Functional leg length	Anthropometer (Seca, Hamburg, Germany)
Foot length	Segmometer (Cescorf, Porto Alegre, Brazil)

^a^ Measuring range: 7–99 cm, Graduation: 1 mm.

^b^ Measuring range: 0–300 cm, Graduation: 1 mm.

A process of calibration will be performed in preparation for the experiment to establish the global coordinate system. Fifty-three (53) reflective markers (Figure 2; Table 3) will be placed on the subject’s UE and LE respectively using skin-friendly double-sided tapes. Participants will be instructed to hold a static pose in the standard anatomical position to define the skeleton and the JCS.

The PROM measurements will precede the AROM measurements so that the participants become familiar with the movements. The PROM movements will be carried out by the researcher involved in the study. Subjective information such as pain will be recorded and any deviation from the recommended testing position due to discomfort will be described in the comments (refer to Appendix 1) (Menabde et al., 2017). A medical treatment table will be used for performing the movements, which will be placed in the cameras’ field of view. Participants will be instructed to relax the muscles associated with the movement during the PROM trials to avoid potential impact on the ROM. The determination of the end-feel will be carried out slowly and carefully to avoid any muscle or joint injuries. Two assisted trials of each joint motion will be performed at a comfortable speed for the participant with a 2-s pause at the maximal PROM. In succession to the assisted trials, two consecutive unassisted trials of each motion will be performed by the participant to determine the AROM. Each movement will be repeated twice to ensure the maximum ROM is reached. The sequence of the movements will be highly dependent on the experimental time allotment, hence the movements requiring supine starting position will be performed consecutively followed by prone and seated positions (Table 5). The time required to perform both PROM and AROM measurements is estimated to be 20 min. The following subsections provide detailed illustrations of the movements adopted in this study. The movements presented in this protocol are adapted from “Musculoskeletal assessment: joint range of motion, muscle testing, and function” by Clarkson (2020) and “Measurement of joint motion: a guide to goniometry” by Norkin and White (2016).

**TABLE 5:** Classification of movements based on the starting positions.

Joint	Starting position		
	Supine	Prone	Seated
Shoulder	Flexion Abduction Internal rotation External rotation	Extension	
Elbow	Flexion Extension		Pronation Supination
Wrist			Flexion Extension Radial deviation Ulnar deviation
Hip	Flexion Abduction Adduction	Extension	Internal rotation External rotation
Knee	Flexion Extension		Internal rotation External rotation
Ankle			Dorsiflexion Plantarflexion Inversion Eversion

#### 3.3.1 The shoulder

**FIGURE 3:**
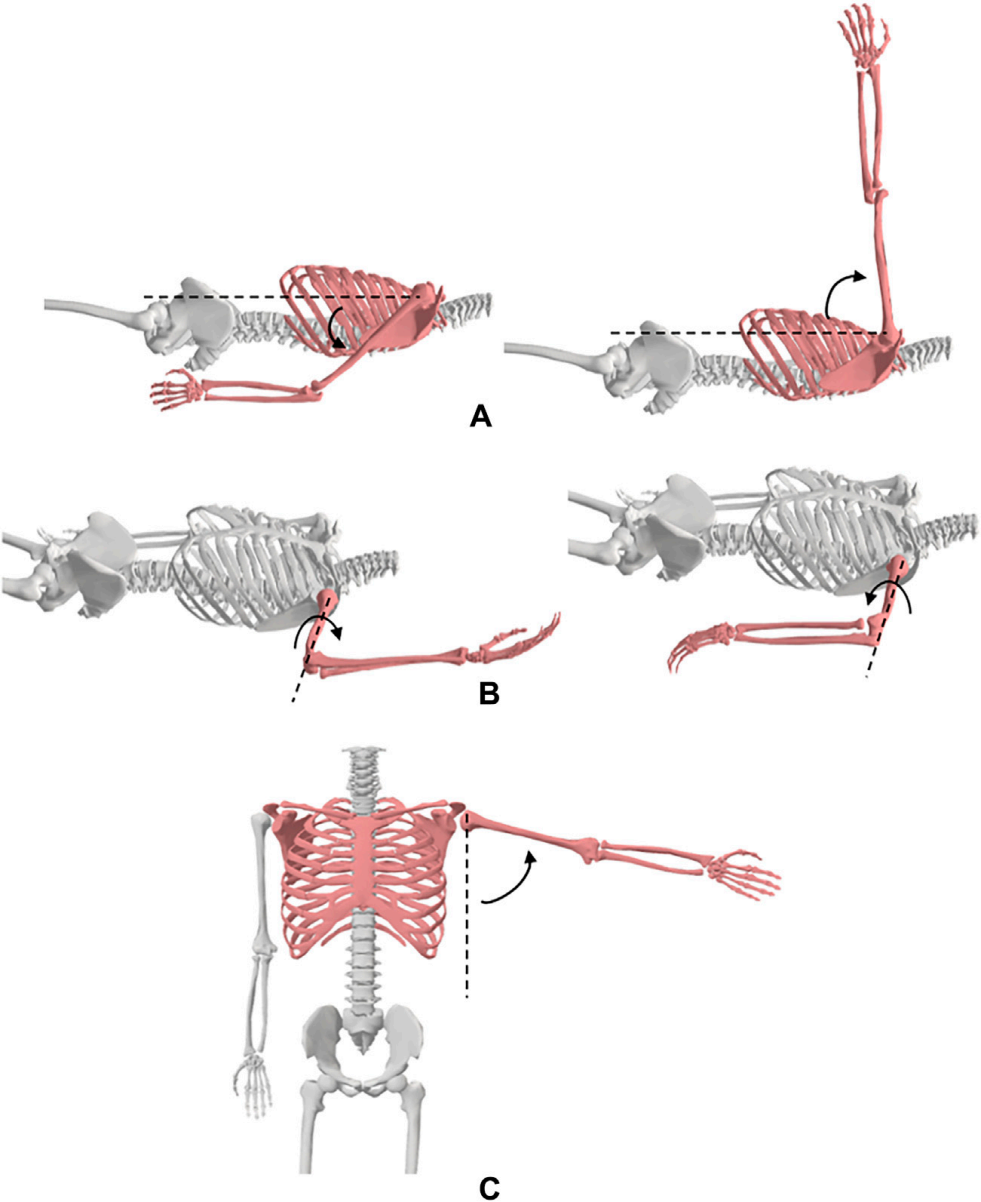
Shoulder movements; **(A)** flexion and extension, **(B)** medial and lateral rotation, **(C)** abduction.

**Motion 1:** Flexion/Extension (Figure 3A). **Starting position:** For flexion, the subject will be positioned in the supine anatomical position. For extension, the subject will be positioned prone with his/her shoulder at 0° and elbow slightly flexed. The motions can also be performed in seated starting positions. **Procedure:** The shoulder is flexed by lifting the humerus, allowing for a slight rotation to attain maximum flexion. The shoulder is extended by lifting the humerus while maintaining neutral adduction/abduction.

**Motion 2:** Adduction/Abduction (Figure 3C). **Starting position:** For abduction, the subject will be positioned in the supine anatomical position. For adduction, the subject will be positioned in the supine position with his/her shoulder at 90° flexion. The motions can also be performed in seated starting positions. **Procedure:** The shoulder is abducted by moving the arm laterally while maintaining lateral rotation and neutral extension/flexion. The shoulder is adducted by moving the humerus medially while maintaining shoulder flexion and internal rotation.

**Motion 3:** Medial/Lateral Rotation (Figure 3B). **Starting position:** The subject will be positioned in the supine position with his/her arm at 90° shoulder abduction and elbow at 90° flexion such that the palm of the hand faces downwards. **Procedure:** The shoulder is medially rotated by moving the forearm downwards while maintaining shoulder abduction and elbow flexion. For lateral rotation, the forearm is moved posteriorly bringing the dorsal surface of the palm downwards while maintaining shoulder abduction and elbow flexion.

#### 3.3.2 The elbow

**FIGURE 4:**
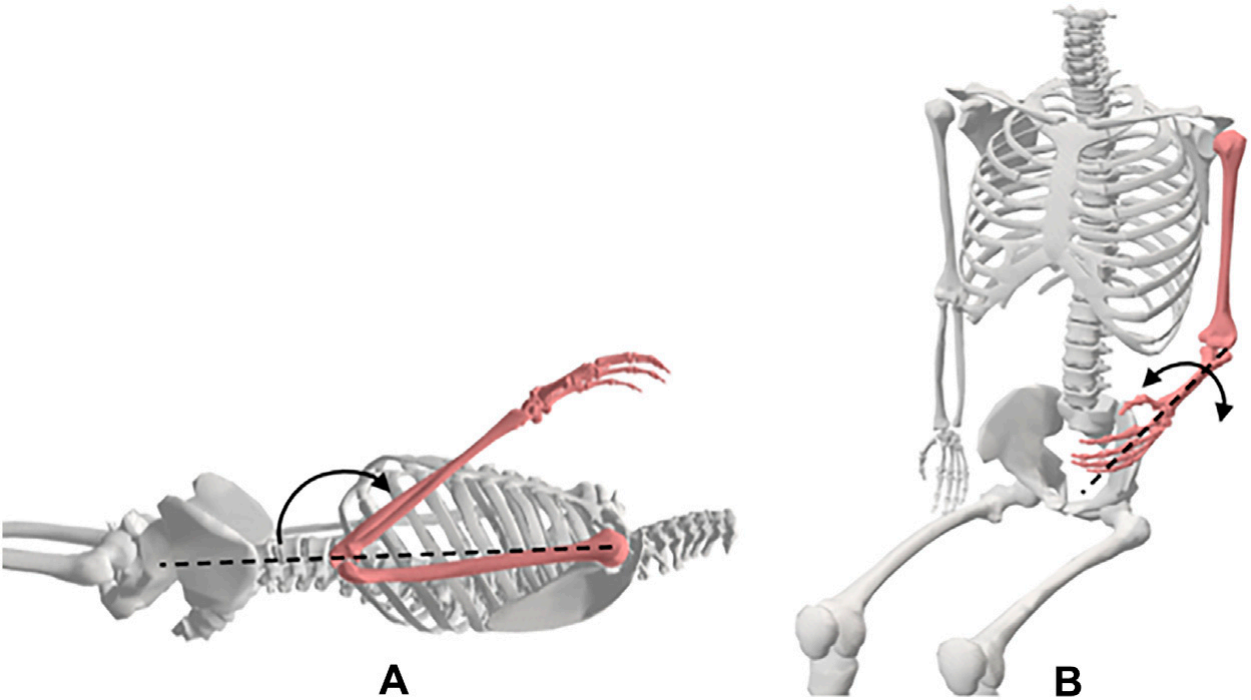
Elbow movements; **(A)** flexion and extension, **(B)** forearm pronation and supination.

**Motion 1:** Flexion/Extension (Figure 4A). **Starting position:** For flexion, the subject will be positioned in the supine anatomical position. A towel roll will be placed under the distal end of the humerus to allow for full elbow extension. The elbow maximum extension will be considered the starting position of elbow flexion. The motions can also be performed in seated starting positions. **Procedure:** The elbow is flexed by moving the hand towards the shoulder while maintaining forearm supination.

**Motion 2:** Pronation/Supination of the forearm (Figure 4B). **Starting position:** The subject will be placed in the seated position with his/her shoulder in 0° flexion and abduction, and elbow flexed to 90°. The forearm will be positioned midway between supination and pronation such that the thumb is pointing upwards. **Procedure:** The forearm is pronated by rotating the hand such that the palm faces downwards. The arm is supinated by rotating the hand such that the palm faces upwards.

#### 3.3.3 The wrist

**FIGURE 5:**
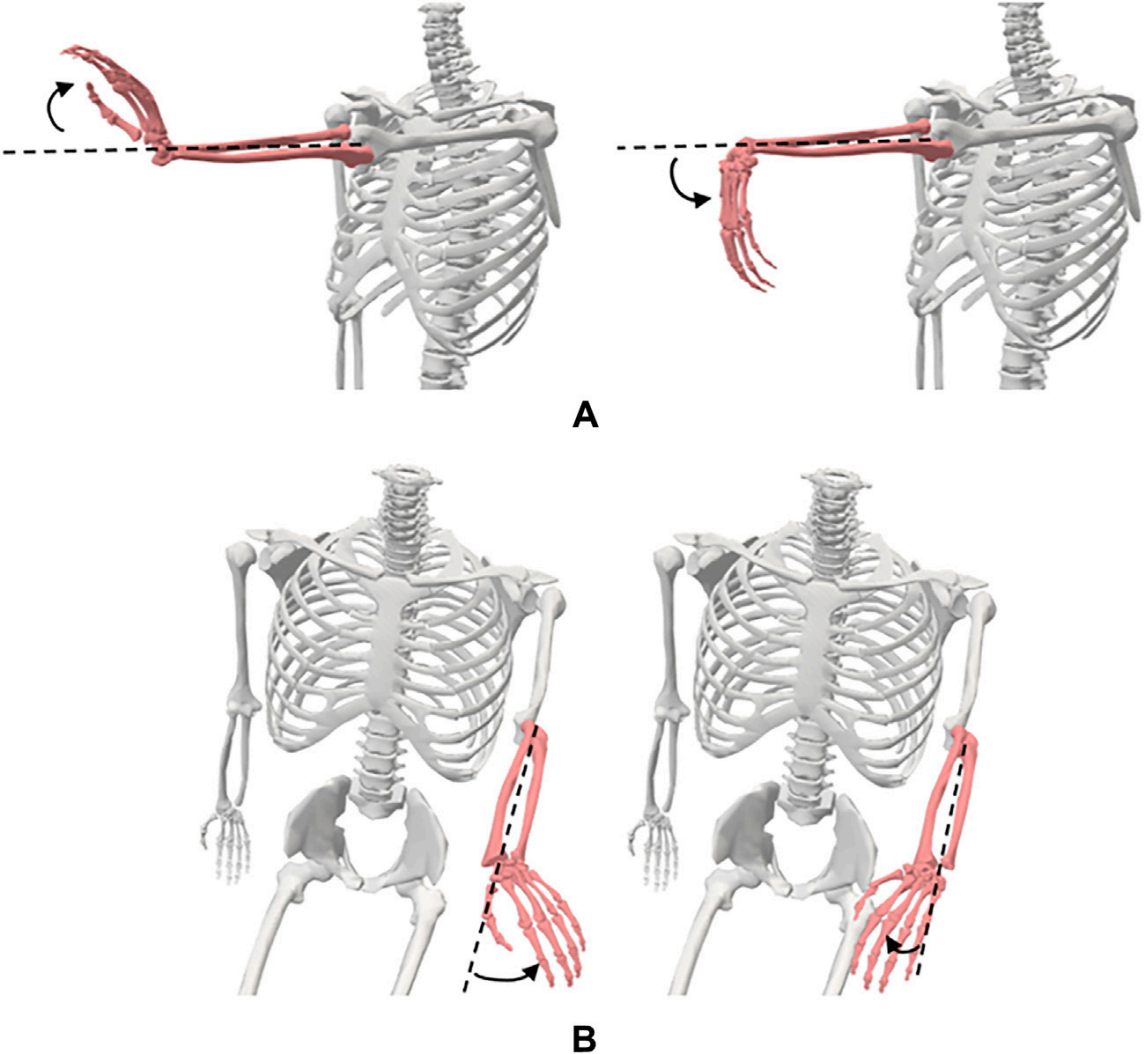
Wrist movements; **(A)** flexion and extension, **(B)** radial and ulnar deviation.

**Motion 1:** Flexion/Extension (Figure 5A). **Starting position:** For flexion and extension, the subject will be positioned seated next to a supporting surface with his/her shoulder at 90° abduction and elbow 90° flexion. The forearm will be placed on a surface with the palm facing downwards. **Procedure:** The wrist is flexed by pushing on the dorsal surface of the hand moving it downwards. The wrist is held at 0° of ulnar and radial deviation. The wrist is then extended by pushing the palm surface of the hand, moving it upwards. The wrist is held at 0° of ulnar and radial deviation.

**Motion 2:** Radial/Ulnar Deviation (Figure 5B). **Starting position:** The subject will be positioned in a seated position next to a supporting surface with his/her shoulder at 90° abduction and elbow 90° flexion. The forearm will be placed on a surface with the palm facing downwards. **Procedure:** The wrist is radially deviated by moving the hand towards the thumb while maintaining the wrist at 0° of flexion and extension. The wrist is deviated in the ulnar direction by moving the hand towards the little finger while maintaining the wrist at 0° of flexion and extension.

#### 3.3.4 The hip

**FIGURE 6:**
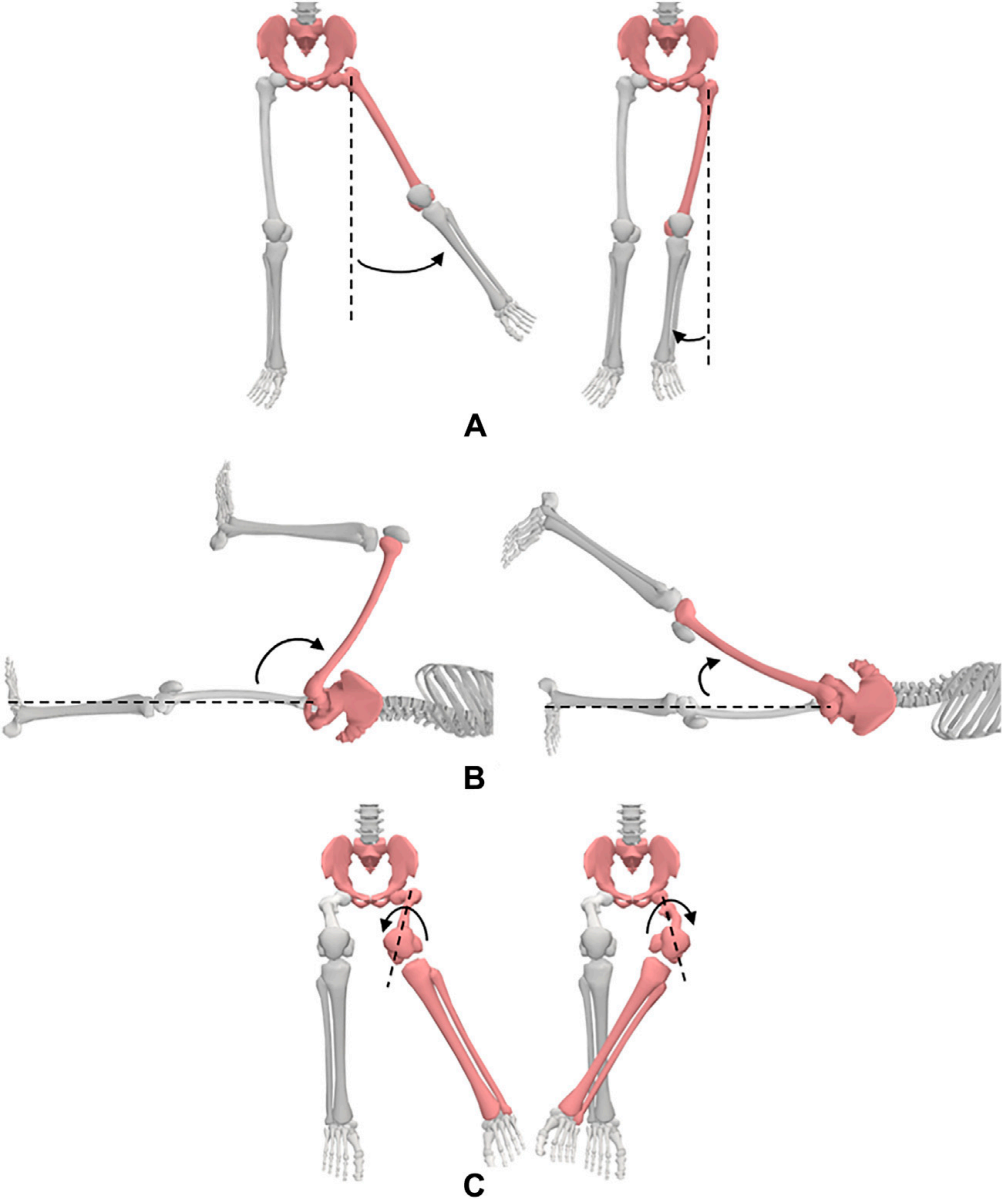
Hip movements; **(A)** abduction and adduction, **(B)** flexion and extension, **(C)** medial and lateral rotation.

**Motion 1:** Flexion/Extension (Figure 6B). **Starting position:** For flexion, the subject will be placed in the supine position with his/her pelvis in the neutral position, knee extended, and both hips in 0° of abduction, adduction, and rotation. For extension, the subject will be placed in the prone position with both of his/her knees extended, and hips in 0° abduction, adduction, and rotation. **Procedure:** The hip is flexed by lifting the thigh off the table while allowing the knee to flex passively during the motion. The extremity is maintained in neutral abduction, adduction, and rotation. The hip is extended by raising the LE from the table while maintaining knee extension throughout the motion.

**Motion 2:** Adduction/Abduction (Figure 6A). **Starting position:** The subject will be placed in the supine position with his/her knees extended and hips in 0° of flexion, extension, and rotation. **Procedure:** The hip is abducted by moving the LE laterally while maintaining 0° flexion, extension, and rotation of the hip. The hip is adducted by moving the LE medially towards the other extremity while maintaining 0° flexion, extension, and rotation of the hip.

**Motion 3:** Medial/Lateral Rotation (Figure 6C). **Starting position:** The subject will be positioned seated on a firm surface with his/her knees flexed to 90° over the edge of the surface. The hip will be placed in 0° of abduction and adduction and 90° flexion. A towel will be placed beneath the distal end of the femur. **Procedure:** For medial rotation, one hand is placed at the distal femur for stabilization and the other hand is used to move the shank laterally from the distal tibia. For lateral rotation, one hand is placed at the distal femur for stabilization and the other hand is used to move the shank medially from the distal tibia.

#### 3.3.5 The knee

**FIGURE 7:**
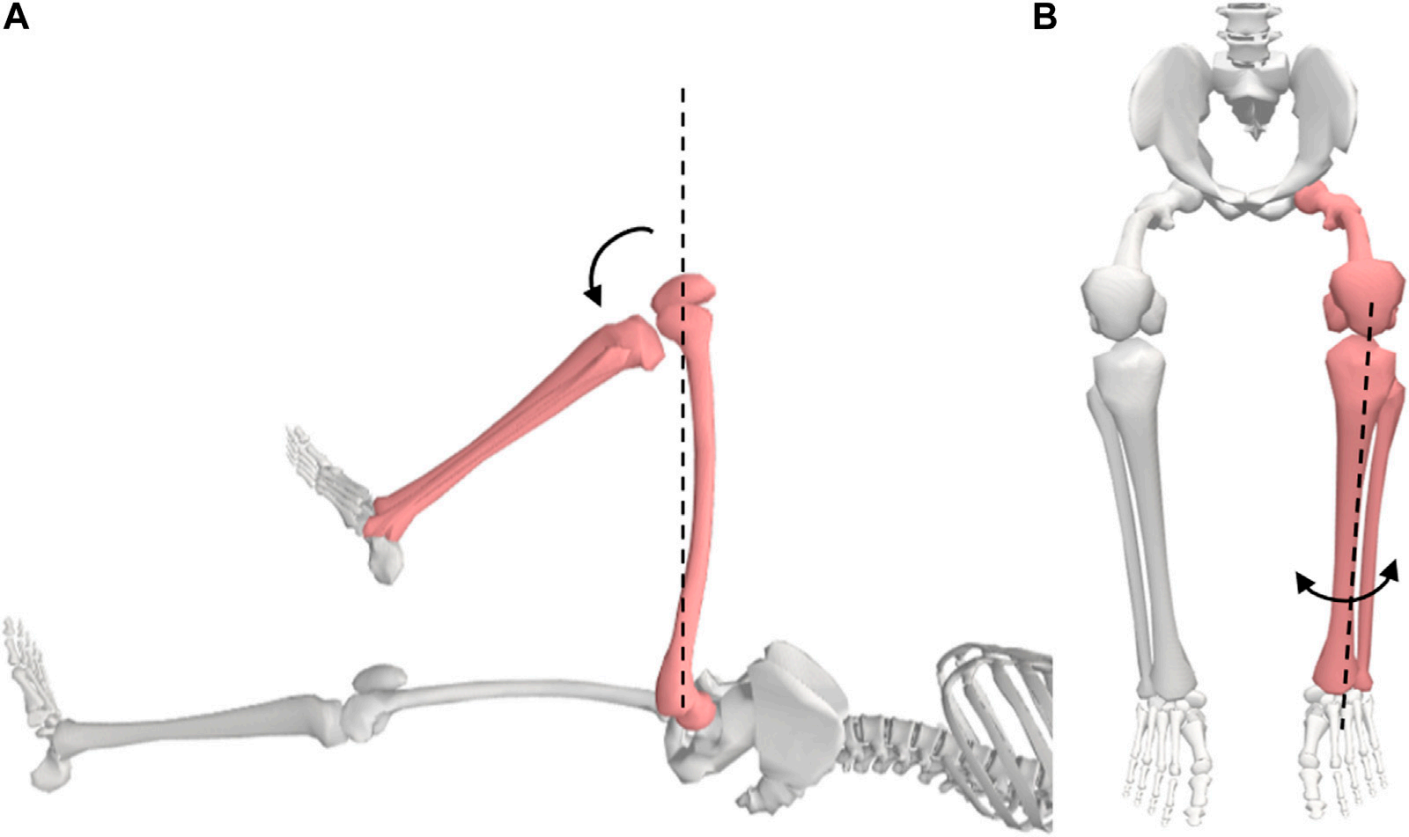
Knee movements; **(A)** flexion, **(B)** medial and lateral rotation.

**Motion 1:** Flexion/Extension (Figure 7A). **Starting position:** For flexion, the subject will be placed in the supine position with his/her knee extended, hip in 0° of extension, abduction, and adduction. A towel will be placed under the ankle to allow maximum knee extension. Knee extension is recorded as the starting position for knee flexion. **Procedure:** To flex the knee, the ankle is held with one hand and the anterior thigh with the other hand. The subject’s thigh is moved to approximately 90° of hip flexion and his/her knee moved into flexion.

**Motion 2:** Medial/Lateral Rotation (Figure 7B). **Starting position:** The subject will be positioned seated with his/her knee flexed to 90° with a towel beneath the lower thigh. **Procedure:** The tibia is rotated internally and externally through the full ROM.

#### 3.3.6 The ankle

**FIGURE 8:**
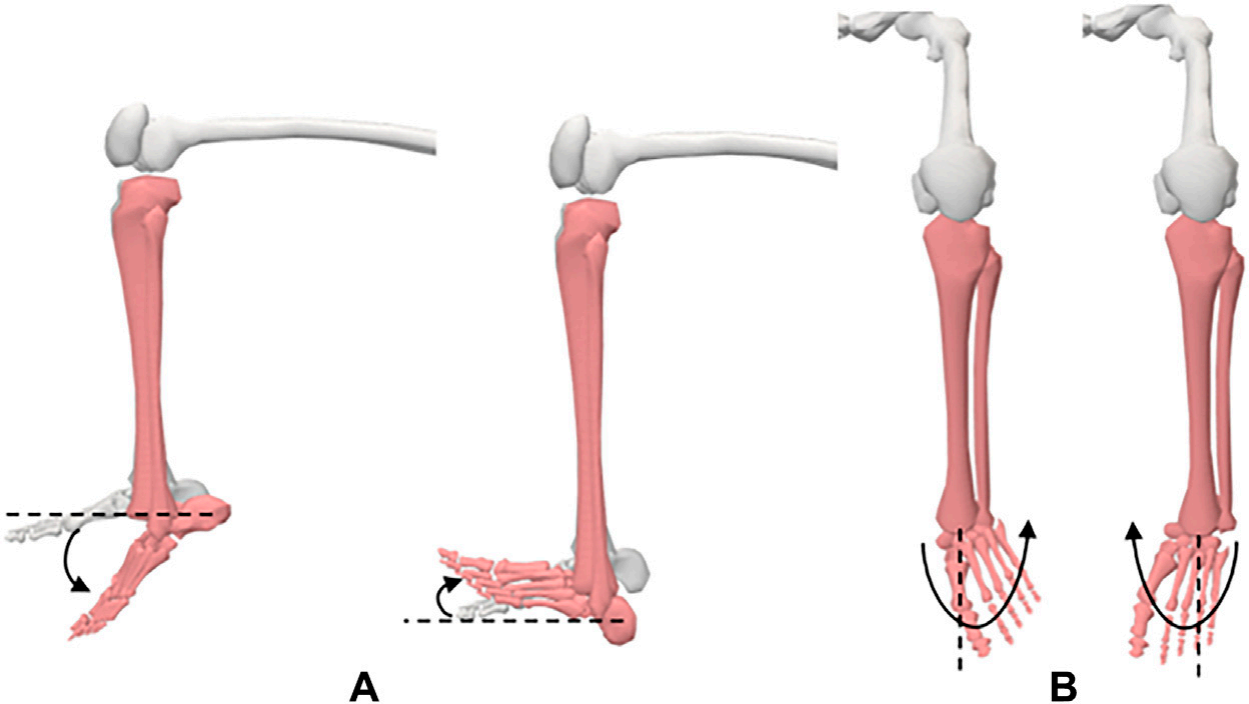
Ankle movements; (**A**) dorsiflexion and plantarflexion, (**B**) inversion and eversion.

**Motion 1:** Plantarflexion/Dorsiflexion (Figure 8A). **Starting position:** The subject will be placed in a seated position with his/her knee flexed to 90°. His/her foot should be in 0° of inversion and eversion. **Procedure:** The foot is moved into dorsiflexion by rotating the foot, moving the top of the foot upwards. The foot is rotated bringing the bottom of the foot downwards to produce plantarflexion.

**Motion 2:** Inversion/Eversion (Figure 8B). **Starting position:** The subject will be placed in a seated position with his/her knee flexed to 90°. **Procedure:** For inversion, the forefoot is moved medially into adduction and downward into plantarflexion such that the medial side of the foot is higher than the lateral side of the foot. For eversion, the forefoot is moved laterally into abduction and upward into dorsiflexion such that the lateral side of the foot is higher than the medial side of the foot.

## 4 Outcome measures

Anonymised/pseudonymised marker data and outcome measures will be recorded via secure data servers according to the data protection laws for analysis.

In the proposed study, the marker trajectories will be recorded over the whole motion and will be used to define the skeleton and the JCS. Defining the skeleton and the JCS facilitates the graphical representation of the joint kinematics of the movements. For the purpose of the proposed study, only the minimum and maximum points of the angle waveform are of interest, i.e., the highest point on the shoulder flexion/extension curve represents the maximum shoulder flexion, and the minimum represent the maximum extension. A single maximum and minimum will be extracted from both the PROM and AROM trials to represent the maximum ROM. The data will be reported in the table shown in appendix 1, which includes the level of discomfort/pain sustained by the participants for every joint motion.

Descriptive statistical analysis (mean, standard deviation, frequencies, quartiles, etc.) will be conducted to characterize the study sample and ROM parameters. The participants would be characterized based on the Chi-squared test. Correlation analysis will be performed to examine the relationship between demographic and anthropometric variables with ROM parameters. Significance level would be set at 5%.

## 5 Discussion

The protocol described in this paper is only applicable in MA labs with 3D MA systems. The protocol includes the JCS along with the segment definitions to describe the rotation of one segment about the joint axis with respect to a stationary reference segment. The marker set presented in this paper includes markers selected based on the ISB recommendations as well as necessary markers to define the anatomical segments and facilitate the joint angle calculation using the Cortex software. Slight variations in the proposed marker set are justifiable for future studies according to the motion analysis software as well as the equipment utilized to measure and record the joints’ ROM. The movements adopted in this study have been selected from various sources to incorporate all joint rotations while ensuring the isolation of each joint motion during the movements. It is recommended that future studies utilize this protocol to draw a relationship between the joints’ ROM and the adjacent segments characteristics, i.e., segment length, joint stiffness, etc.

While 3D MA techniques are commonly used in quantifying dynamic motion (Ore et al., 2020; Reissner et al., 2019; Lempereur et al., 2014; Tojima et al., 2013), errors due to soft tissue artefacts (STA) present a major challenge (Cutti et al., 2005; Slater et al., 2018). For example, dynamic movements such as running or jumping cause skin movements which can alter the relative displacement between markers and the underlying bone resulting in errors in the calculation of the joint kinematics (Cutti et al., 2005). Several methods have been adopted to compensate for some components of STAs such as the global optimization method (GOM) which is based on numerical simulations (Lu and O’connor, 1999), and the use of additional markers at the joints to experimentally perform corrections based on knowledge of plausible joint motions (Schmidt et al., 1999). Although our proposed study involves measuring the maximum ROM of the UE and LE joints in children in static positions, STA are still present even when there is no dynamic motion (Hara et al., 2014). Hence, we will study the benefit of GOM on the data obtained from the proposed study. Additionally, the results of the proposed cross-sectional study will be validated with normative data.

## Data Availability

The raw data supporting the conclusions of this article will be made available by the authors, without undue reservation.
