# Supplementary material for: A protocol for obtaining upper and lower extremity joints’ range of motion in children using three-dimensional motion analysis system
Source: Front Physiol. 2024 Aug 21;15:1416175. doi: 10.3389/fphys.2024.1416175 (PMC11371791; doi:10.3389/fphys.2024.1416175)
Supplement: Supplementary file 1 [file DataSheet1.pdf]

Anthropometric Measurements and Range of Motion Assessment Form

Participant's Name ______________________ **Age** ____________

Examiner Name ______________________ **Date** ____________

Anthropometric Measurements

Segment	Length
Bideltoid breadth	
Biacromial breadth	
Shoulder-Elbow length	
Elbow-Hand length	
Bi-trochanter breadth	
Buttock-Knee length	
Knee height	
Functional leg length	

*Segment length in cm

Additional notes: ______________________

Range of Motion Assessment

Joint	Motion	AROM	Pain Level	PROM	Pain Level	Comments
Shoulder	Flexion					
	Extension					
	Abduction					
	Medial rotation					
	Lateral rotation					
Elbow	Flexion					
	Extension					
	Pronation					
	Supination					
Wrist	Flexion					
	Extension					
	Radial deviation					
	Ulnar deviation					
Hip	Flexion					
	Extension					
	Abduction					
	Adduction					
	Medial rotation					
	Lateral rotation					
Knee	Flexion					
	Extension					
	Medial rotation					
	Lateral rotation					
Ankle	Plantarflexion					
	Dorsiflexion					
	Inversion					
	Eversion					

*AROM and PROM measurements in degrees

*Pain level indicated by a pain scale from 0 – 10, where 0 indicates no pain and 10 indicates unbearable pain

Pain Assessment Tool

PAIN ASSESSMENT CHART

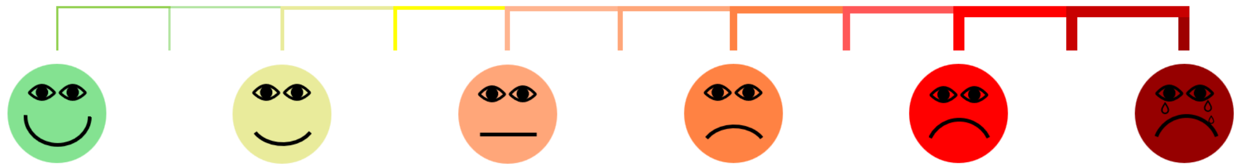

NO PAIN	MILD PAIN	MODERATE PAIN	SEVERE PAIN	VERY SEVERE PAIN	WORST PAIN POSSIBLE
لا يوجد ألم	ألم بسيط	ألم متوسط	ألم شديد	ألم شديد جدا	ألم لا يطاق
